# Optimized Balloon-Expandable Transcatheter Aortic Valve Implantation and Clinical Outcomes

**DOI:** 10.1016/j.jacadv.2026.103037

**Published:** 2026-08-26

**Authors:** Luka Vitez, Domenico Angellotti, Daijiro Tomii, Jonas Lanz, Daryoush Samim, Cyril Ferro, Ottavia Cozzi, Nadir Elamin, Joanna Bartkowiak, Fabien Praz, David Reineke, Stefan Stortecky, Stephan Windecker, Thomas Pilgrim

**Affiliations:** a Department of Cardiology, Inselspital, Bern University Hospital, University of Bern, Bern, Switzerland; b Department of Cardiac Surgery, Inselspital, Bern University Hospital, University of Bern, Bern, Switzerland; c Department of Cardiology, Bürgerspital Solothurn, Solothurn, Switzerland

**Keywords:** depth, implantation coaxiality, TAVR optimization, valve expansion

## Abstract

**Background:**

The expansion of transcatheter aortic valve replacement (TAVR) to younger patients with longer life expectancy requires procedural optimization to enhance long-term valve durability.

**Objectives:**

To evaluate the relationship between fluoroscopic determinants of optimal transcatheter heart valve (THV) implantation—coaxiality, implantation depth, and expansion—and clinical and valve-related outcomes.

**Methods:**

In this cohort study, consecutive patients undergoing transfemoral TAVR with balloon-expandable prostheses (Sapien S3, Sapien S3 Ultra) between February 2014 and June 2022 were included. THV coaxiality, implantation depth, and expansion were assessed in the 3-cusp view following deployment using standardized protocols. Suboptimal implantation was defined as ≥1 unfavorable determinants (coaxiality ≥4.8°, depth ≥6 mm, or underexpansion ≥20%). Outcomes included the modified early safety endpoint, moderate or severe hemodynamic valve deterioration, and stage 2 bioprosthetic valve failure.

**Results:**

Among 1,032 patients (mean age 81 ± 6 years, 35.1% female), suboptimal THV implantation occurred in 443 (42.9%) patients and was driven by noncoaxial deployment (n = 240, 23.3%), excessive implantation depth (n = 261, 25.3%), and underexpansion (n = 54, 5.2%). Suboptimal implantation was associated with a higher likelihood of the modified early safety endpoint at 30 days (43.3% vs 19.9%; adjusted odds ratio: 3.05; 95% CI: 2.30-4.04) and stage 2 bioprosthetic valve failure at 5 years (subHR: 4.40; 95% CI: 1.25-13.59). Patients with ≥2 determinants demonstrated higher rates of hemodynamic valve deterioration at 5 years (subHR: 3.48; 95% CI: 1.24-9.80).

**Conclusions:**

Optimal THV implantation was associated with improved early procedural safety and long-term THV durability in balloon-expandable TAVR. Systematic fluoroscopic assessment of optimized valve implantation may enhance patient outcomes.

Transcatheter aortic valve replacement (TAVR) has evolved into a standard therapy for elderly patients with severe aortic stenosis.^1^ With the expansion to younger patients, increasing attention has shifted toward refining implantation techniques to further enhance procedural success and valve durability.^2^^,^^3^ In this context, intraprocedural fluoroscopic assessment of optimal transcatheter heart valve (THV) deployment has emerged as a central focus. Recently, a structured framework for the conceptual evaluation of 4 key elements—THV coaxiality, orientation (commissural alignment), depth, and expansion (CODE)—has been proposed, thereby standardizing the immediate postprocedural evaluation of valve implantation.^4^ These parameters are strongly influenced by patient-specific anatomic characteristics, implantation techniques, as well as device-related factors.^5^ Optimizing them is clinically relevant, as suboptimal valve deployment may predispose patients to paravalvular regurgitation, conduction disturbances requiring permanent pacemaker implantation (PPI), impaired coronary access, and potentially accelerated structural valve deterioration.^6^, ^7^, ^8^, ^9^, ^10^ To our knowledge, no prior study has comprehensively evaluated the integrated impact of coaxiality, depth, and expansion on early procedural safety and long-term durability in contemporary balloon-expandable valves. Accordingly, we sought to systematically assess these fluoroscopic determinants of optimal THV implantation and examine their association with short-term safety and long-term clinical outcomes in patients undergoing TAVR with a balloon-expandable prostheses.

## Methods

### Population

We performed a retrospective analysis of prospectively collected data from the Bern TAVI Registry which is part of the SwissTAVI Registry (NCT01368250). The registry was approved by the local ethics committee, and all patients provided written informed consent for participation. Consecutive patients treated at Bern University Hospital with transfemoral TAVR for severe native aortic stenosis using either the balloon-expandable SAPIEN 3 or SAPIEN 3 Ultra valve (Edwards Lifesciences) were included. High-quality contrast aortography and a standardized three-cusp projection following valve deployment were required to allow precise assessment of prosthesis angulation, implantation depth relative to the native annulus, and valve expansion. Exclusion criteria were nontransfemoral access, valve-in-valve procedures, technical failure as defined by Valve Academic Research Consortium-3 (VARC-3) criteria (including procedural death, procedural vascular stent implantation, conversion to surgery, and valve-in-series or repositioning for valve dislocation/embolization), TAVR performed for pure aortic regurgitation, missing echocardiographic follow-up, implantation of a 20-mm prosthesis, and inadequate fluoroscopic imaging.^11^

### Data collection

All baseline clinical, procedural, and follow-up data were prospectively recorded in a web-based database maintained by the Clinical Trials Unit at the Department of Clinical Research of the University of Bern. Standardized baseline echocardiographic and computed tomographic (CT) imaging datasets were independently re-evaluated by experienced imaging specialists and subsequently added to the registry. Device landing zone calcium volume was quantified on contrast-enhanced CT using a predefined threshold of 850 Hounsfield units, as previously validated.^12^ Aortic valve disease was assessed by transthoracic echocardiography performed by experienced operators, and the severity of aortic stenosis was determined according to current guidelines recommendations using peak velocity, mean gradient, and aortic valve area calculated by the continuity equation.^1^^,^^13^ Echocardiographic follow-up was recommended at discharge, 30 days, 1 year, and 5 years after TAVR, with additional examinations performed when clinically indicated. Both planned and unplanned echocardiograms were prospectively documented using standardized case report forms. Clinical follow-up at 30 days and 5 years was obtained through structured interviews, documentation from referring physicians, and hospital discharge records. THV sizing was retrospectively reassessed based on the implanted prosthesis in relation to CT-derived annular area or perimeter.^14^ Oversizing and undersizing were defined according to manufacturer recommendations as >5% deviation above or below the recommended annular range. All clinical events were reviewed in a blinded manner by a dedicated clinical event committee after evaluation of original source documents. Independent central data monitoring by the Clinical Trials Unit ensured data completeness and accuracy.

### Fluoroscopic assessment of optimal THV deployment

Angiograms were independently reviewed by 4 investigators (L.V., D.A., N.E., and C.F.) under blinded conditions regarding all clinical data and outcomes. Determinants of interest regarding optimal THV deployment included valve coaxiality, implantation depth, and expansion. Suboptimal deployment was defined as the presence of at least one unfavorable determinant. Orientation, defined as rotational alignment of the commissural posts relative to the native commissures, was not assessed due to the absence of standardized fluoroscopic metrics in balloon-expandable valves.

### THV coaxiality

Assessment of THV coaxiality was performed fluoroscopically using previously described methods.^6^^,^^15^ In brief, noncontrast cine angiographic images in the 3-cusp view obtained following valve deployment were retrospectively analyzed. A single frame with the least-motion artifacts was selected to measure the angle between the line connecting the nadirs of the native cusps and the line intersecting the lower hinge points of the prosthesis outflow frame. Coaxiality was defined as the angle between the native annular plane and the prosthetic valve frame. Unfavorable coaxiality was recorded if the angle was ≥4.8°.^15^

### THV expansion

Expansion was assessed using the commissural post–height method, which has been previously described and validated against CT.^16^ After calibration, THV diameter was measured at the mid-portion of the stent frame, and expansion was calculated as the percentage difference between the measured and nominal diameters. According to the manufacturer, nominal diameters were 22.75, 25.71, and 28.75 mm for the 23-, 26-, and 29-mm SAPIEN 3/SAPIEN 3 Ultra valves, respectively.^17^ Underexpansion was recorded if the percentage difference between the measured and the nominal diameter was ≥20%.^6^

### Implantation depth

Depth was measured in the 3-cusp view following THV deployment as the distance between the lower edge of the stent frame to the nadir of the noncoronary cusp, consistent with prior studies.^18^^,^^19^ The threshold for suboptimal implantation depth was derived using receiver operating characteristic curve analysis with modified device early safety, and values ≥6 mm were considered suboptimal.

### Outcomes of interest

The outcomes of interest included both short- and long-term endpoints. The short-term outcome was modified device early safety at 30 days after TAVR. Long-term outcomes included stage 2 bioprosthetic valve failure (BVF) and modified hemodynamic valve deterioration (HVD) at 5 years, as well as all-cause mortality, cardiovascular mortality, and cerebrovascular events at 5 years. Modified valve early safety was defined as freedom from: 1) all-cause mortality; 2) any cerebrovascular event; 3) major or life-threatening bleeding according to VARC-2; 4) major vascular or cardiac structural complications; 5) acute kidney injury stage 2 or 3; 6) moderate or severe aortic regurgitation; 7) new PPI; and 8) device-related reintervention at 30 days. Stage 2 BVF was defined as any transcatheter or surgical reintervention related to the prosthetic device, excluding patients with endocarditis. Modified HVD was defined as: 1) transprosthetic gradient ≥20 mm Hg and increase of ≥10 mm Hg detected ≥90 days after TAVR compared with echocardiographic assessment at discharge or 30 days after TAVR; or 2) a new occurrence or an increase of ≥1 grade of transprosthetic aortic regurgitation resulting in moderate or greater aortic regurgitation ≥90 days after TAVR.

### Statistical analysis

Normality was assessed using the Shapiro–Wilk test. Categorical variables are presented as counts and percentages and compared using the chi-square test. Continuous variables are expressed as mean ± SD or median (IQR), as appropriate, and compared using the Student’s *t*-test or Mann–Whitney U test. Associations with the early safety endpoint were evaluated using logistic regression, with results reported as ORs and 95% CIs. Variables included in the regression models were selected based on clinical rationale and comprised valve size, age, sex, and the Society of Thoracic Surgeons Predicted Risk of Mortality (STS-PROM) score. Multivariable analysis of predictors of suboptimal THV implantation was performed using logistic regression with adjustment for age, sex, valve size, body mass index, and STS-PROM score. Calibration of logistic regression models was assessed using the Hosmer–Lemeshow goodness-of-fit test. Temporal changes in suboptimal THV implantation were assessed by dividing the study period into tertiles according to implantation date and formally tested using logistic regression with implantation years as a continuous predictor. Fine-Gray regression analysis was used to estimate subdistribution hazard ratios (subHRs) for stage 2 BVF and modified HVD, with death treated as a competing event. Because of the limited number of events, multivariable adjustment for modified HVD and stage 2 BVF was restricted to age and valve size to avoid model overfitting. Cox proportional-hazards models were used to estimate hazard ratios (HRs) with 95% CIs for all-cause death, cardiovascular death, cerebrovascular events, and HVD. Models for all-cause death, cardiovascular death, and cerebrovascular events were adjusted for valve size, age, sex, and STS-PROM score. Death was not treated as a competing event in the analysis of cerebrovascular events. Individual determinants of suboptimal THV implantation were assessed for their association with HVD using separate univariable Cox models and a multivariable Cox model including noncoaxiality, implantation depth, and underexpansion. The proportional hazards assumption for Cox regression models was assessed using Schoenfeld residuals and was not violated. The optimal implantation depth for predicting PPI was identified using the Youden index. Implantation depth classified new pacemaker implantation with an AUC of 0.66 (95% CI: 0.61-0.71; *P* < 0.001). A cutoff of 6 mm yielded a sensitivity of 56% and specificity of 70% and was used for the purpose of the present analysis. Cutoff values for coaxiality and expansion were adopted from previously published studies using the same dataset of patients.^6^^,^^15^ Pairwise associations between individual determinants of suboptimal THV implantation were assessed using Phi coefficients and demonstrated weak correlations. Collinearity diagnostics showed no evidence of problematic multicollinearity (all variance inflation factors <2), supporting the use of a cumulative categorical variable reflecting the number of suboptimal deployment determinants. For time-to-event analyses, follow-up time was calculated from the date of the index TAVI procedure to the first occurrence of the endpoint of interest. Patients who did not experience the endpoint were censored at the time of last available follow-up, or at the end of the study period. Missing-data patterns were assessed using Little’s missing completely at random test. Complete-case analysis was performed for regression models, and patients with missing data were excluded from the denominator for percentage calculations. Variables with missing values exceeding 30% were excluded from the analysis. Missing data for each variable is shown in Supplemental Table 1. All tests were two-sided with a significance level of *P* < 0.05. Analyses were performed using R for Mac 2025.09.2 (R Foundation for Statistical Computing).

## Results

**Figure 1:**
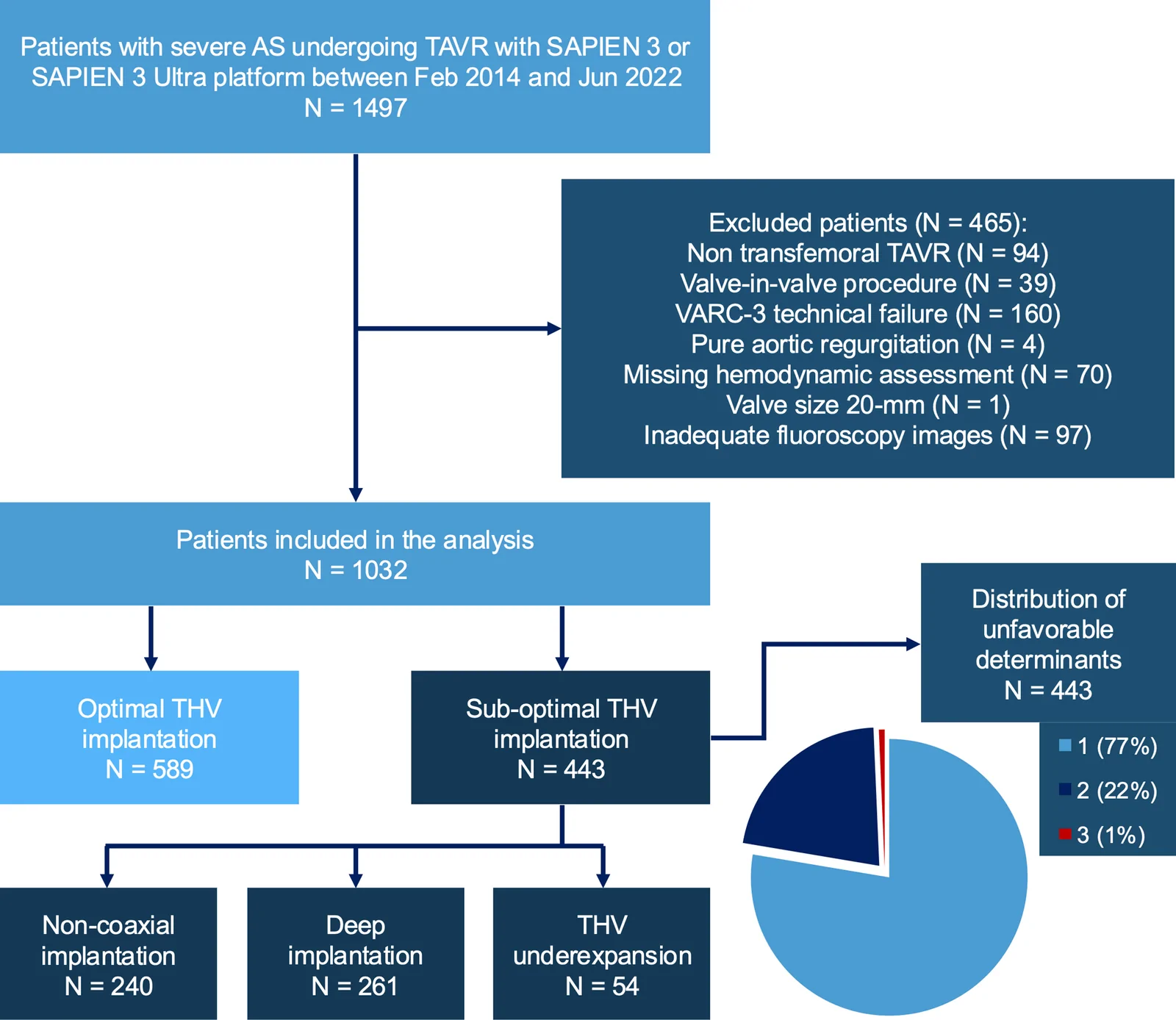
**Study Flowchart** Flow diagram illustrating included/excluded patients and the distribution of unfavorable determinants of coaxiality, depth, and expansion. AS = aortic stenosis; TAVR = transcatheter aortic valve replacement; THV = transcatheter heart valve; VARC-3 = Valve Academic Research Consortium 3.

Among 2,780 patients who underwent TAVR for severe aortic stenosis at Bern University Hospital between February 2014 and June 2022, a total of 1,497 (53.8%) received a SAPIEN 3 or SAPIEN 3 Ultra balloon-expandable THV. After excluding patients who underwent nontransfemoral TAVR (n = 94), valve-in-valve procedures (n = 39), experienced VARC-3 technical failure (n = 160), had pure aortic regurgitation (n = 4), lacked echocardiographic follow-up (n = 70), received a 20-mm prosthesis (n = 1), or had inadequate fluoroscopic imaging (n = 97), a total of 1,032 patients remained for the present analysis. The mean age of the patients was 81 ± 6 years, and 361 were women (35.1%). The median STS-PROM was 3.2% (IQR: 2.0-5.0). Bicuspid aortic valve anatomy was present in 4.9% of the population (Table 1). Noncoaxial implantation was present in 240 patients (23.3%), underexpansion in 54 patients (5.2%), and depth of implantation ≥6 mm in 261 patients (25.3%). Overall, 443 patients (42.9%) had at least one determinant of suboptimal valve implantation, 96 patients (9.3%) had 2, and 3 patients (0.2%) had 3 determinants of suboptimal valve implantation (Figure 1).

**Table 1:** Baseline Characteristics

	Overall (N = 1,032)	Optimal THV Implantation (n = 589)	Suboptimal THV Implantation (n = 443)	*P* Value
Age, y (SD)	81 (6)	81 (6)	81 (6)	0.78
Women (%)	361 (35.0)	211 (35.8)	150 (33.9)	0.51
BMI, kg/m^2^ (IQR)	27 (24-30)	26.4 (23.1-30.0)	26.7 (24.3-30.0)	0.16
STS PROM (IQR)	3.2 (2.0-5.0)	3.2 (2.0-5.0)	3.3 (1.9-5.0)	0.92
Arterial hypertension (%)	905 (87.7)	519 (88.1)	389 (87.8)	0.88
Diabetes (%)	292 (28.4)	169 (28.7)	126 (28.4)	0.93
Dyslipidemia (%)	735 (71.4)	429 (72.8)	309 (69.8)	0.28
COPD (%)	88 (8.6)	47 (8.0)	41 (9.3)	0.47
eGFR, mL/min/1.73 m^2^ (SD)	58 (24)	55 (20)	59 (21)	0.28
Coronary artery disease (%)	594 (57.7)	346 (58.7)	251 (56.7)	0.50
Peripheral artery disease (%)	65 (6.3)	43 (7.3)	22 (5.0)	0.13
Atrial fibrillation (%)	305 (29.6)	174 (29.5)	134 (30.2)	0.81
Previous cerebrovascular event (%)	109 (10.6)	61 (10.4)	48 (10.8)	0.80
Previous cardiac surgery (%)	76 (7.4)	39 (6.6)	37 (8.4)	0.29
Previous pacemaker implantation (%)	74 (7.2)	49 (8.3)	25 (5.6)	0.10
Baseline echocardiography				
LVEF ≤40% (%)	144 (13.9)	61 (10.3)	83 (18.7)	**<0.001**
Mean gradient, mm Hg (IQR)	41 (30-48)	41 (30-48)	40 (29-49)	0.10
AVA, cm^2^ (IQR)	0.7 (0.6-0.9)	0.8 (0.6-0.9)	0.8 (0.6-0.9)	0.94
iAVA, cm^2^/m^2^ (IQR)	0.28 (0.23-0.32)	0.28 (0.23-0.32)	0.27 (0.22-0.33)	0.96
AR ≥2	79 (7.7)	46 (7.8)	33 (7.5)	0.83
MR ≥2	131 (12.7)	67 (11.4)	64 (14.4)	0.11
TR ≥2	82 (7.9)	37 (6.2)	45 (10.2)	**0.02**
Computed tomography				
Valve morphology (%)				0.96
Bicuspid	51 (4.9)	29 (4.9)	22 (5.0)	
Tricuspid	870 (84.3)	498 (84.6)	372 (84.0)	
Annulus area, mm^2^ (IQR)	464 (416-544)	455 (411-530)	487 (425-555)	**0.002**
LVOT area, mm^2^ (IQR)	429 (349-524)	408 (343-513)	447 (367-567)	**<0.001**
Aortic angulation, ° (IQR)	51 (45-57)	50 (44-57)	52 (46-58)	**0.017**
Annulus eccentricity (SD)	0.77 (0.06)	0.77 (0.06)	0.77 (0.06)	0.99
Total aortic valve calcium, mm^3^ (IQR)	246 (111-442)	267 (212-447)	219 (107-428)	0.08
Total LVOT calcium, mm^3^ (IQR)	0 (0-2.4)	0 (0-2.2)	0 (0-3.0)	0.33

Statistically significant *P* values (*P* < 0.05) are in **bold**.

AR = aortic regurgitation; AVA = aortic valve area; BMI = body mass index; COPD = chronic obstructive pulmonary disease; eGFR = estimated glomerular filtration rate; iAVA = indexed aortic valve area; IQR = interquartile range; LVEF = left ventricular ejection fraction; LVOT = left ventricular outflow tract; MR = mitral regurgitation; SD = standard deviation; STS PROM = Society of Thoracic Surgeons Predicted Risk of Mortality; THV = transcatheter heart valve; TR = tricuspid regurgitation.

### Clinical characteristics

Reduced left ventricular ejection fraction (LVEF ≤40%) and moderate or greater tricuspid regurgitation at baseline were more frequent in patients with suboptimal THV implantation than in those with optimal THV implantation (61/589, 10.3% vs 83/443 18.7%; *P* < 0.001; and 37/589, 6.2% vs 45/443, 10.2%; *P* = 0.02). Patients with suboptimal THV implantation had a larger annulus area (455 mm^2^ [IQR: 411-530] vs 487 mm^2^ [IQR: 425-555]; *P* = 0.002) and greater aortic angulation (50° [IQR: 44-57] vs 52° [IQR: 46-58]; *P* = 0.017). No statistically significant differences were observed regarding comorbidities or other echocardiographic or CT-derived parameters.

### Procedural characteristics

Procedural characteristics are presented in Table 2. Patients with suboptimal THV implantation more frequently received a 29-mm prosthesis than those with optimal THV implantation (23.1% vs 37.0%; *P* < 0.001). This valve size had also a higher prevalence of oversizing (4.1% vs 11.2%; *P* = 0.010). The rate of predilatation and postdilatation did not differ across groups. Implantation of a larger valve (29 mm) and reduced LVEF (≤40%) were identified as independent predictors of suboptimal THV implantation (Table 3). The Hosmer–Lemeshow test did not indicate poor fit for the multivariable logistic regression model (*P* = 0.30). When the study population was divided into tertiles according to implantation date, the incidence of suboptimal THV implantation increased over time, from 122/346 (35.3%) in the earliest tertile to 152/342 (44.4%) and 169/344 (49.1%) in the second and latest tertiles, respectively. In logistic regression, later implantation was associated with higher odds of suboptimal implantation (OR per 1-year increase 1.09; 95% CI: 1.03-1.15; *P* = 0.002). In parallel, predilatation rates decreased significantly across implantation-date tertiles, from 225/346 (65.0%) to 183/342 (53.5%) and 112/344 (32.6%), respectively (*P* < 0.001; *P* for trend <0.001).

**Table 2:** Procedural Characteristics

	Overall (N = 1,032)	Optimal THV Implantation (n = 589)	Suboptimal THV Implantation (n = 443)	*P* Value
Valve size (%)				**<0.001**
23 mm	238 (23.1)	155 (26.3)	83 (18.7)	
26 mm	494 (47.9)	298 (50.6)	196 (44.2)	
29 mm	300 (29.1)	136 (23.1)	164 (37.0)	
Oversizing	234 (22.7)	120 (20.4)	114 (25.7)	**0.042**
Oversizing according to valve size				
23 mm	47 (4.6)	27 (4.6)	20 (4.5)	0.22
26 mm	113 (10.9)	69 (11.7)	44 (9.9)	0.86
29 mm	74 (7.2)	24 (4.1)	50 (11.2)	**0.010**
Undersizing	77 (7.5)	51 (8.7)	26 (5.9)	0.09
Predilatation	520 (50.4)	305 (51.8)	215 (48.5)	0.30
Postdilatation	145 (14.1)	92 (15.6)	53 (12.0)	0.09
Procedure time, min	40 (31-51)	39 (31-50)	41 (31-51)	0.19
Procedural complications (%)				
Vascular complications	104 (10.1)	58 (9.8)	46 (10.4)	0.78
Cardiac tamponade	5 (0.5)	3 (0.5)	2 (0.5)	0.90
Hemodynamic instability	8 (0.8)	4 (0.7)	4 (0.9)	0.69
Coronary artery occlusion	3 (0.3)	2 (0.3)	1 (0.2)	0.74
Annular rupture or aortic dissection	8 (0.8)	5 (0.8)	3 (0.7)	0.76
Discharge echocardiography				
AVA, cm^2^ (IQR)	1.6 (1.4-1.9)	1.6 (1.4-1.9)	1.6 (1.3-1.9)	0.06
Mean gradient, mm Hg (IQR)	7 (4-10)	7 (4-10)	7 (4-9)	0.17
Mean gradient ≥20 mm Hg (%)	17 (1.6)	11 (1.9)	6 (1.4)	0.52
≥ Moderate PVR	14 (1.4)	7 (1.2)	7 (1.5)	0.60

Statistically significant *P* values (*P* < 0.05) are in **bold**.

AVA = aortic valve area; PVR = paravalvular regurgitation.

**Table 3:** Multivariable Analysis of Predictors of Suboptimal THV Implantation

	aOR	95% CI	*P* Value
Age per 1-year increase	0.99	0.96-1.02	0.52
Female	0.67	0.43-1.02	0.06
BMI per 1 kg/m^2^ increase	1.02	0.99-1.05	0.26
STS PROM per 1% increase	1.02	0.95-1.08	0.62
Aortic angulation per 1◦ increase	1.01	0.99-1.02	0.55
LVEF ≤40%	1.59	1.02-2.48	**0.041**
TR ≥2	1.78	0.97-3.28	0.07
Oversizing >5%	1.45	0.99-2.13	0.06
Valve size			
26 vs 23 mm	1.50	0.93-2.40	0.10
29 vs 23 mm	2.77	1.55-4.95	**<0.001**
Postdilatation	0.86	0.54-1.38	0.54

Statistically significant *P* values (*P* < 0.05) are in **bold**.

BMI = body mass index; LVEF = left ventricular ejection fraction; STS PROM = Society of Thoracic Surgeons Predicted Risk of Mortality; TR = tricuspid regurgitation.

### Short-term outcomes

At 30 days, patients with suboptimal THV implantation experienced significantly higher rates of the modified early safety endpoint than those with optimal valve implantation (192/443, 43.3% vs 117/589, 19.9%; aOR: 3.05; 95% CI: 2.30-4.04; *P* < 0.001) (Central Illustration). This difference was driven primarily by a higher need for PPI in the suboptimal group (28.7% vs 3.7%; aOR: 9.57; 95% CI: 5.94-15.4; *P* < 0.001). The likelihood of the modified early safety composite outcome increased progressively with a greater number of suboptimal THV implantation determinants: compared with optimal THV implantation, 1 and 2 to 3 determinants of suboptimal THV implantation were associated with progressively greater odds of adverse events (1 vs 0; aOR: 2.92; 95% CI: 2.06-4.15; *P* < 0.001, 2-3 vs 0; aOR: 10.21; 95% CI: 5.90-17.66; *P* < 0.001, and 2-3 vs 1; aOR: 2.89; 95% CI: 1.82-4.66; *P* < 0.001). The Hosmer–Lemeshow test did not indicate poor fit for the logistic regression models evaluating the modified early safety endpoint (*P* = 0.23). Rates of cerebrovascular events, VARC-2 major or life-threatening bleeding, major vascular or cardiac complications, VARC-2 acute kidney injury stage 2 to 3, moderate or greater paravalvular regurgitation, and reintervention did not differ statistically significantly between groups (Table 4).

**Central Illustration:**
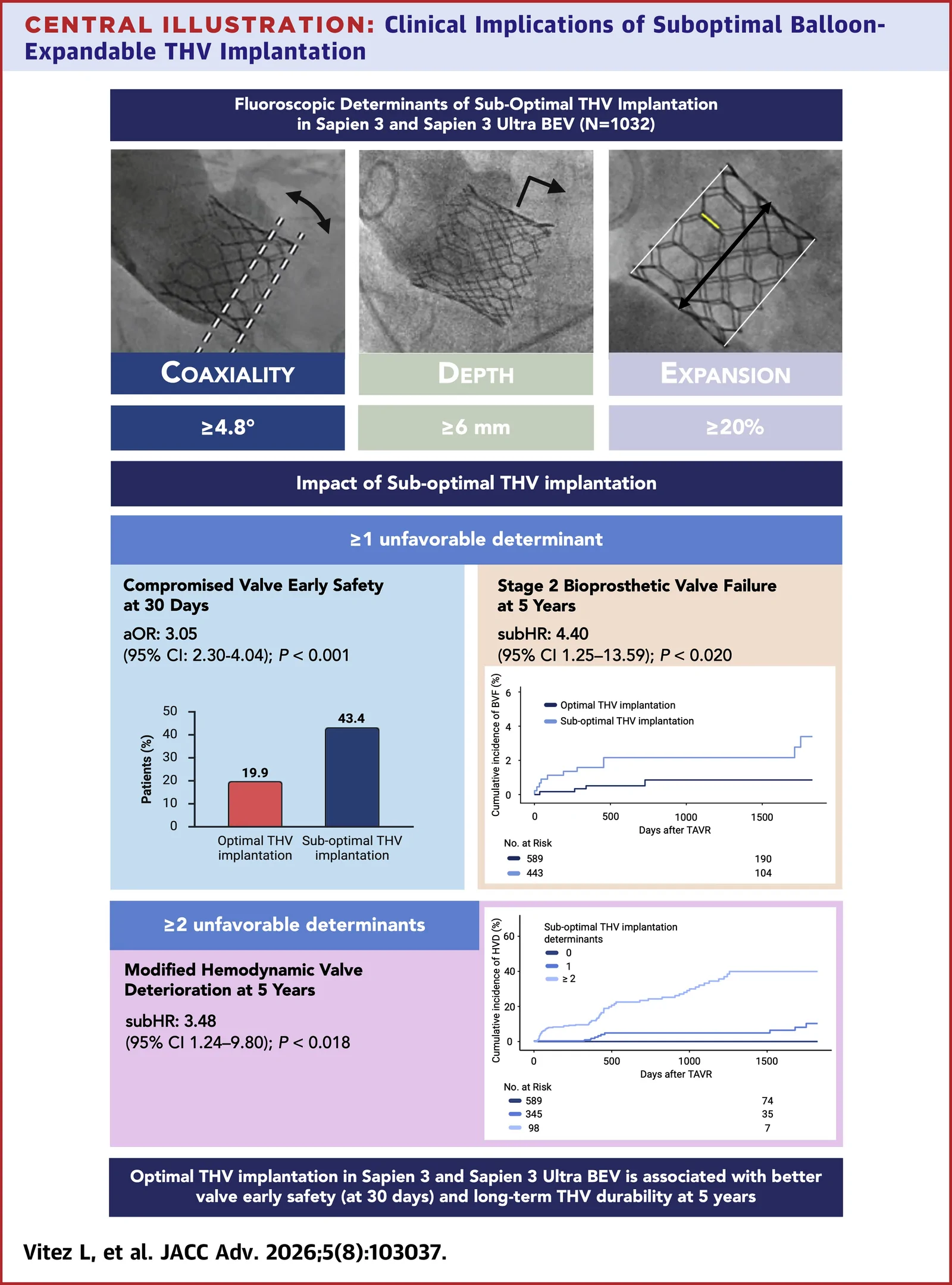
**Clinical Implications of Suboptimal Balloon-Expandable THV Implantation** Optimal transcatheter heart valve (THV) implantation is associated with better early valve safety and long-term valve durability in Sapien 3 and Sapien 3 Ultra balloon-expandable valves (BEVs). aOR = adjusted OR; subHR = subdistribution HR; TAVR = transcatheter aortic valve implantation.

**Table 4:** Early Clinical Outcomes

	Optimal THV Implantation (n = 589)	Suboptimal THV Implantation (n = 443)	OR/aOR 95%CI *P* Value
Compromised early safety—30 d	117 (19.9)	192 (43.3)	3.05 2.30-4.04 **<0.001**
All-cause mortality	0	0	-
Cerebrovascular events	19 (3.2)	14 (3.2)	1.08 0.53-2.21 0.82
VARC-2 major or life-threatening bleeding	66 (11.2)	57 (12.9)	1.16 0.79-1.71 0.45
Major vascular or cardiac complication	69 (11.7)	53 (12.0)	1.03 0.70-1.51 0.90
AKI stage 2 to 3	6 (1.0)	2 (0.5)	0.44 0.09-2.19 0.32
≥ moderate PVR	7 (1.2)	7 (1.6)	1.33 0.46-3.82 0.60
PPI	22 (3.7)	127 (28.7)	9.57 5.94-15.4 **<0.001**
Reintervention	0	2 (0.5)	-

Statistically significant *P* values (*P* < 0.05) are in **bold**.

AKI = acute kidney injury; PPI = permanent pacemaker implantation; PVR = paravalvular regurgitation; THV = transcatheter heart valve; VARC = Valve Academic Research Consortium.

### Long-term outcomes

During a median follow-up of 389 days (IQR: 365-1,789), stage 2 BVF occurred in 16/1,032 patients (1.6%). Reinterventions consisted of surgical valve replacement in 9/16 patients, balloon valvuloplasty in 4/16 patients, and redo-TAVI in 3/16 patients. Suboptimal THV implantation was associated with a higher cumulative incidence of stage 2 BVF compared with optimal implantation (12/443, 2.7% vs 4/589, 0.7%; subHR: 4.40; 95% CI: 1.25-13.59; *P* = 0.020). The use of smaller prostheses (23-mm valves) was a significant predictor of stage 2 BVF (23 mm vs 26 or 29 mm; subHR: 3.58; 95% CI: 1.35-9.52; *P* = 0.011). Although older age appeared to be associated with a lower risk of stage 2 BVF (subHR per 1-year increase: 0.92; 95% CI: 0.88-0.96; *P* < 0.001), there was no significant interaction between age and suboptimal THV implantation (*P* = 0.96) (Table 5, Central Illustration).

**Table 5:** Long-Term Clinical Outcomes: Stage 2 Bioprosthetic Valve Failure and Hemodynamic Valve Deterioration at 5 Years

	subHR	95% CI	*P* Value
Stage 2 BVF			
Suboptimal THV implantation	4.40	1.25-13.59	**0.020**
Age (per year)	0.92	0.88-0.96	**<0.001**
Valve size: 23 vs ≥ 26 mm	3.58	1.35-9.52	**0.011**
HVD			
1 vs 0 determinants	1.48	0.72-3.04	0.29
2 or 3 vs 0 determinants	3.48	1.24-9.8	**0.018**
Age (per year)	0.93	0.88-0.98	**0.008**
Valve size: 23 vs ≥ 26 mm	4.42	2.21-8.84	**<0.001**

Statistically significant *P* values (*P* < 0.05) are in **bold**.

BVF = bioprosthetic valve failure; HVD = hemodynamic valve deterioration; subHR = subdistribution hazard ratio; THV = transcatheter heart valve.

**Figure 2:**
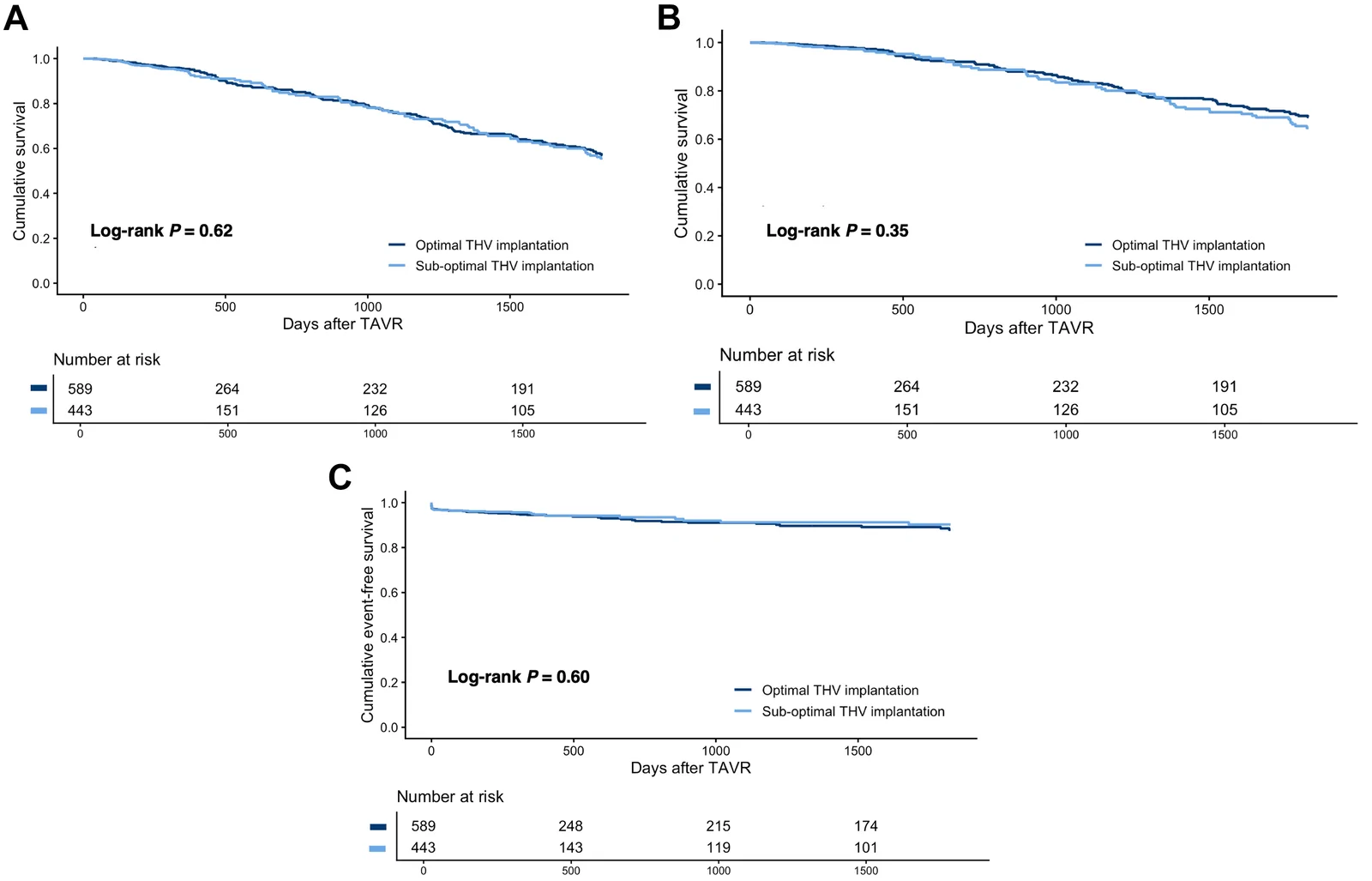
**Survival Outcomes at 5 Years** Kaplan-Meier curves for all-cause mortality (A), cardiovascular mortality (B), and cerebrovascular events (C) at 5 years after transcatheter aortic valve replacement (TAVR) for optimal and suboptimal transcatheter heart valve (THV) implantation.

Modified HVD occurred in 28 of 1,032 patients (2.7%) overall, including 14/589 (2.3%) in the optimal and 14/443 (3.2%) in the suboptimal THV implantation group. The etiology of HVD was an increased transprosthetic gradient in 23/28 patients, significant intraprosthetic regurgitation in 1/28, and a mixed mechanism in 4/28. The presence of at least 2 determinants of suboptimal THV implantation was associated with a higher risk of modified HVD at 5 years compared to patients with optimal THV implantation (subHR: 3.48; 95% CI: 1.24-9.80; *P* = 0.018) (Table 5, Central Illustration). The use of smaller prostheses (23-mm valve) was significantly associated with an increased risk of modified HVD (subHR: 4.42; 95% CI: 2.21-8.84; *P* < 0.001). Among the individual determinants of suboptimal THV implantation, underexpansion was the strongest predictor of HVD (HR: 6.82; 95% CI: 2.99-15.55; *P* < 0.001), whereas noncoaxiality and deep implantation were not significantly associated with this endpoint. In a multivariable model including all 3 determinants, underexpansion remained independently associated with HVD (aHR: 6.61; 95% CI: 2.85-15.32; *P* < 0.001) (Supplemental Table 2). Rates of all-cause mortality, cardiovascular mortality, and cerebrovascular events were comparable between groups (Table 6, Figure 2).

**Table 6:** Long-Term Clinical Outcomes: Mortality and Cerebrovascular Events at 5 Years

	Optimal THV Implantation (n = 589)	Suboptimal THV Implantation (n = 443)	aHR 95% CI *P* Value
All-cause mortality	139 (23.5)	89 (20.2)	0.92 0.71-1.21 0.55
Cardiovascular mortality	89 (15.0)	61 (13.9)	0.98 0.71-1.36 0.91
Cerebrovascular events	47 (8.0)	29 (6.5)	0.94 0.59-1.51 0.80

aHR = adjusted HR; THV = transcatheter heart valve.

## Discussion

With advances in valve design, expansion of TAVR indications, and refined implantation techniques, optimal THV implantation represents an important procedural objective that contributes to favorable long-term outcomes.^3^^,^^20^ In this retrospective study of 1,032 balloon-expandable TAVR patients, suboptimal THV implantation, as defined by coaxiality, implantation depth, and valve expansion, occurred in 43% of cases and was associated with a greater likelihood of the modified early safety endpoint and long-term stage 2 BVF. These findings underscore the importance of systematic intraprocedural assessment of valve implantation and suggest that optimal THV deployment represents a modifiable determinant of both short-term and long-term valve performance.

Potentially modifiable procedural factors influencing clinical outcomes of TAVI were previously consolidated within the CODE framework, a structured tool to fluoroscopically assess CODE of THV.^4^ Our findings align with and expand upon prior work evaluating CODE components in isolation. Noncoaxial deployment and underexpansion have been increasingly recognized as contributors to impaired valve hemodynamics and accelerated degeneration.^21^^,^^22^ Excessive leaflet or annular calcification, a horizontal aortic root, and suboptimal sizing can all lead to incomplete expansion, which is linked to higher residual gradients, increased leaflet stress, subclinical leaflet thrombosis, and earlier structural valve deterioration.^22^, ^23^, ^24^, ^25^ Consistent with these mechanisms, we observed that the accumulation of unfavorable fluoroscopic determinants generated an adverse geometric configuration associated with a nearly 3.5-fold higher risk of HVD and a 4.5-fold higher incidence of stage 2 BVF at 5 years.

Pacemaker implantation after TAVR has been linked to adverse long-term outcomes, including increased mortality.^8^^,^^26^ Excessively deep valve deployment is a known contributor to irreversible conduction system injury by mechanical compression.^18^^,^^19^ In our cohort, the overall PPI rate of 14.4% is consistent with previous reports and confirms a strong association between deep implantation at the noncoronary cusp and PPI.^27^ Using a data-driven threshold, implantation depth ≥6 mm was associated with a higher likelihood of PPI, supporting its relevance as a marker of suboptimal deployment. This relationship was further amplified when multiple unfavorable fluoroscopic determinants were present, resulting in a 9.5-fold increased likelihood for PPI.

Valve size demonstrated opposing effects on early and late outcomes: Larger prostheses (29 mm) were linked to suboptimal implantation and higher PPI rates, whereas smaller valves (23 mm) were associated with greater HVD and reintervention. Consistent with these findings, 29-mm valves were associated with a higher rate of oversizing, suggesting that appropriate valve sizing is particularly important in patients with larger anatomies. These findings reflect distinct biomechanical mechanisms and raise important questions regarding the need for size-specific optimization strategies, including systematic assessment of expansion and selective use of post-dilatation.^5^^,^^6^ Notably, predilatation and postdilatation rates did not differ significantly between optimal and suboptimal implantation groups, suggesting that these techniques were either not major determinants of implantation quality in this cohort or were not used selectively or frequently enough to measurably influence valve hemodynamics or, potentially, long-term outcomes. Although LVOT calcium, annular eccentricity, and total valve calcium burden did not differ significantly between optimal and suboptimal implantation groups, patients with suboptimal implantation had greater aortic angulation and larger annular/LVOT dimensions. These findings suggest that device–anatomy interaction may play an important role in larger and more angulated anatomies, where coaxial alignment, deployment stability, and final frame geometry may be more challenging to achieve. Interestingly, suboptimal implantation increased over time as predilatation rates declined. Although causality cannot be inferred, this temporal association raises the hypothesis that less-frequent lesion preparation may have contributed to more challenging THV deployment or expansion in selected patients.

Reduced LVEF (≤40%) also emerged as an independent predictor of suboptimal THV implantation. Patients with impaired systolic function represent a higher-risk cohort with more complex procedural characteristics.^28^ In this setting, intraprocedural hemodynamic compromise during positioning and deployment may limit opportunities for optimization. Although older age was associated with a lower incidence of reintervention, formal interaction testing showed no significant interaction between age and suboptimal THV implantation, suggesting that the association between implantation quality and reintervention was not age-dependent. The higher incidence of BVF and HVD reported in younger patients with surgical and transcatheter aortic bioprostheses may partly reflect a greater inflammatory activity and/or more pronounced host-device interaction in this population.^29^^,^^30^

### Clinical implications

Optimal THV implantation is a key modifiable determinant of both early safety and long-term hemodynamic durability in balloon-expandable TAVR. Suboptimal deployment—through noncoaxiality, deep positioning, or underexpansion—was common and markedly increased the risks of PPI and stage 2 BVF. Because these features can be identified and corrected intraprocedurally, systematic attention to implant optimization has direct and immediate clinical relevance.

The CODE framework offers a simple, reproducible method to evaluate THV implantation in real time. Integrating coaxiality, depth, and expansion enables operators to detect suboptimal deployment early and optimize when technically feasible. Adequate lesion preparation (eg, predilatation) and precise device positioning are essential to achieve optimal coaxiality and implantation depth at the time of deployment, whereas valve expansion can be further optimized after deployment (eg, by postdilatation). In addition to its clinical utility, the framework may also serve as a standardized tool for operator training, procedural quality assurance, and comparative research across devices and centers. The layered effect observed with the accumulation of multiple unfavorable determinants further supports the importance of an integrated rather than isolated assessment of implantation quality.

Procedurally, our results underscore the need for tailored deployment strategies according to valve size. Larger prostheses (29 mm) require particular attention to implantation depth and sizing to minimize interaction with the membranous septum and reduce the risk of conduction disturbances. Moreover, precise coaxial alignment is essential to prevent asymmetric ventricular protrusion and excessive septal contact.^15^^,^^31^ In contrast, smaller prostheses (23 mm), which are more prone to underexpansion and residual gradients, may benefit from systematic optimization of frame expansion, such as the recently proposed double-tap technique, and selective postdilatation to mitigate long-term hemodynamic deterioration.^20^^,^^31^^,^^32^

From a forward-looking perspective, advances in valve design that promote symmetric expansion, predictable deployment mechanics, and improved fluoroscopic visualization may help reduce technical variability. Emerging intraprocedural technologies including automated projection selection, 3-dimensional fluoroscopy overlay, and artificial intelligence–assisted deployment guidance have the potential to further enhance implantation precision and reduce the incidence of suboptimal positioning.^33^^,^^34^

Taken together, these findings support a procedural paradigm in which fluoroscopic assessment of THV implantation is recognized as a modifiable driver of early device safety and long-term valve durability. Systematic intraprocedural assessment using structured frameworks such as CODE may offer a practical pathway to optimize outcomes across contemporary TAVR practices.

### Study Limitations

This study has several limitations. First, the retrospective observational design introduces the possibility of unmeasured confounding, which may have influenced both procedural characteristics and clinical outcomes, thereby limiting causal interpretation. Second, fluoroscopic assessments of coaxiality, depth, and expansion are influenced by projection angle, image quality, and frame selection; although standardized protocols and consistent reviewers were used, residual measurement variability cannot be excluded. Third, cutoff values for coaxiality and expansion were adopted from previously published analyses using the same underlying dataset. Although these thresholds were predefined in the present study, their derivation from the same cohort may have introduced optimism bias and influenced effect size estimates. External validation is therefore required before these thresholds can be applied more broadly. Fourth, the number of stage 2 BVF events was relatively small, limiting statistical power for long-term analyses and precluding more-extensive multivariable modeling to avoid overfitting. Fifth, ECG-derived conduction parameters were not available for systematic analysis and may have influenced both the incidence and interpretation of PPI outcomes. Finally, the study reflects the experience of a single high-volume center using balloon-expandable valves, which may limit generalizability to other valve platforms, operators with different implantation strategies, or centers with lower procedural volumes. Accordingly, these findings should be considered hypothesis-generating and require prospective validation in broader multicenter populations.

## Conclusions

Optimal THV deployment is an important procedural determinant of both early procedural safety and long-term hemodynamic durability in balloon-expandable TAVR. These findings underscore the importance of systematic intraprocedural assessment of optimal THV implantation and support the integration of structured evaluation tools, such as the CODE framework, into contemporary TAVR practice. As TAVR expands into younger populations, ensuring precise, reproducible deployment will be essential for maximizing valve performance, durability, and long-term patient outcomes.

Perspectives **COMPETENCY IN MEDICAL KNOWLEDGE:** In patients undergoing TAVR, fluoroscopic assessment of THV coaxiality, implantation depth, and expansion provides clinically meaningful information. Suboptimal THV implantation, defined by ≥1 unfavorable determinant, is independently associated with impaired early device safety and increased risk of long-term valve failure. Accumulation of ≥2 unfavorable features substantially increases the risk of hemodynamic valve deterioration. Systematic application of a structured fluoroscopic framework, such as CODE, may improve procedural quality and long-term outcomes.

**TRANSLATIONAL OUTLOOK:** Prospective multicenter studies are needed to validate the CODE framework across different THV platforms, anatomical subsets, and procedural environments. Future development should focus on advanced and automated fluoroscopic imaging techniques to further improve deployment precision. Integration of procedural quality metrics into operator training, performance feedback, and device innovation initiatives may enhance standardization and support continued improvements in TAVR outcomes.

## Funding support and author disclosures

Dr Elamin is a British Cardiac Interventional Society (BCIS) international structural fellow, and his fellowship is funded by Edwards Lifesciences through BCIS and not directly from Edwards Lifesciences. Dr Angellotti is supported by a research grant provided by the CardioPath PhD program. Dr Lanz has received speaker fees to the institution from Edwards Lifesciences and Abbott. Dr Samim received funding for an online course from Edwards Lifesciences. Dr Praz has received travel expenses from Edwards Lifesciences, Abbott Vascular, Medira, Siemens Healthineers, and InQB8 Medical Technologies, as well as a research grant to the institution from Abbott Vascular. Dr Windecker reports research, travel and/or educational grants to the institution from Abbott, Abiomed, Alnylam, Amicus Therapeutics, Amgen, Astra Zeneca, Bayer, B.Braun, Bioanalytica, Biotronik, Boehringer Ingelheim, Boston Scientific, Bristol Myers Squibb, Cordis Medical, CorFlow Therapeutics, CSL Behring, Daiichi Sankyo, Edwards Lifesciences, Fumedica, GE Healthcare, Guerbet, IACULIS, Inari Medical, Janssen AI, Johnson & Johnson, Medalliance, Medtronic, MSD Merck Sharp & Dohme, Neovii Pharmaceutica, Neutromedics AG, Novartis, Novo Nordisk, OM Pharma, Optimapharm, Orchestra BioMed, Pfizer, Philips AG, Sanofi-Aventis, Servier, Shockwave Medical, Siemens Healthcare, Sinomed, SMT Sahajanand Medical Technologies, Vascular Medical, and V-Wave. Dr Windecker serves as advisory board member and/or member of the steering/executive group of trials funded by Abbott, Amgen, Abiomed, Edwards Lifesciences, EnCarda Inc., Medtronic, Novartis, and Sinomed with payments to the institution but no personal payments. He is also a member of the steering/executive committee group of several investigator-initiated trials that receive funding by industry without impact on his personal remuneration. Dr Pilgrim reports research, travel, or educational grant to the institution without personal remuneration from Biotronik, Boston Scientific, Edwards Lifesciences, and ATSens; speaker fees and consultancy fees to the institution from Biotronik, Boston Scientific, Edwards Lifesciences, Abbott, Medtronic, Biosensors, and Highlife. All other authors have reported that they have no relationships relevant to the contents of this paper to disclose.
